# Preoperative low muscle mass is a predictor of falls within 12 months of surgery in patients with lumbar spinal stenosis

**DOI:** 10.1186/s12877-020-01915-y

**Published:** 2020-11-30

**Authors:** Takashi Wada, Shinji Tanishima, Yuki Kitsuda, Mari Osaki, Hideki Nagashima, Hiroshi Hagino

**Affiliations:** 1 Rehabilitation Division, Tottori University Hospital, 36-1 Nishi-cho, Yonago, Tottori, 683-8504 Japan; 2 Division of Orthopedic Surgery, Department of Sensory and Motor Organs, School of Medicine, Tottori University Faculty of Medicine, 36-1 Nishi-cho, Yonago, Tottori, 683-8504 Japan; 3 School of Health Science, Faculty of Medicine, Tottori University, 86 Nishi-cho, Yonago, Tottori, 683-8503 Japan

**Keywords:** Lumbar spinal stenosis, Lumbar spine surgery, Low muscle mass, Fall, Prospective studies

## Abstract

**Background:**

Patients with lumbar spinal stenosis (LSS) may be at high risk of falls due to various factors. No effective fall risk assessments or fall prevention measures have been performed for patients with LSS because only a few studies have evaluated falls in these patients. This study aimed to evaluate the incidence and preoperative predictors of falls within 12 months of surgery in patients with LSS.

**Methods:**

In this prospective study of 82 consecutive preoperative patients with LSS, preoperative demographic data, previous fall history, leg pain, low back pain, Japanese Orthopaedic Association (JOA) score, Hospital Anxiety and Depression Scale (HADS) scores, lower extremity muscle strength, walking speed, grip strength, and muscle mass were assessed at baseline. Falls were assessed at 3, 6, 9, and 12 months after surgery. Participants were categorized as fallers and non-fallers and baseline variables were compared. Binomial logistic regression was used to identify predictors of falls within 12 months of surgery.

**Results:**

Seventy-four patients (90.2%) completed the 12-month follow-up after surgery, of whom 24 patients (32.4%) experienced falls. A higher proportion of fallers were female and had a history of falls compared to non-fallers. Fallers had a significantly lower JOA score and a higher HADS-depression score compared to non-fallers. Fallers had significantly lower tibialis anterior muscle strength, gait speed, grip strength, and skeletal muscle mass index. Fallers had a higher prevalence of low muscle mass compared with non-fallers. The presence of low muscle mass was significantly predictive of falls within 12 months of surgery (odds ratio, 4.46; 95% confidence interval, 1.02–19.63).

**Conclusions:**

Patients with LSS have a high incidence of falls after surgery and preoperative low muscle mass may be a predictor of postoperative falls.

## Background

Falls have been reported to be the main cause of fragility fractures, which increase the burden to society due to increased mortality and economic costs as well as lower quality of life [1, 2]. Many studies on falls were conducted in community-dwelling older people, but some studies were conducted in patients with a musculoskeletal disorder. For example, knee osteoarthritis and osteoporosis have been reported to increase the risk of falls. In patients with musculoskeletal disorders, predictors of falls include muscle weakness and fear of falling [1–3].

Some previous studies on falls have focused on patients with lumbar spinal stenosis (LSS). One study suggested that patients with LSS are at high risk of falling because they experience motor function decline comparable to that of patients with knee osteoarthritis [4]. Another study compared how motor function affects the incidence of falls in patients who underwent surgery and those who did not undergo surgery [5]. These studies did not investigate the actual incidence of falls. In our literature search, we could find only one study reporting the actual incidence of falls in patients with LSS [6]. While this study suggested that poor spinal sagittal balance is associated with postoperative falls, it did not sufficiently adjust for cofounding factors. Another study reported that the risk of hip fracture was higher in patients who underwent lumbar spine surgery compared with those with comparable characteristics (such as age) who did not undergo lumbar spine surgery based on a multivariate analysis [7]. The risk of falls may be higher in patients who undergo lumbar spine surgery because the major cause of hip fracture is falls [8]. We did not identify any studies that prospectively investigated the incidence of falls and conducted multivariate analysis of predictors of falls in patients who have undergone surgery for LSS. Falls in older people are associated with physical factors such as gait speed and muscle weakness as well as psychological factors such as depression [9]. Patients with LSS have slower gait speed, more severe muscle weakness, and poorer psychological condition [10–12]. It has been suggested that these factors may persist after lumbar surgery [13, 14]. Therefore, patients with LSS may be at high risk of falls after surgery. Furthermore, many patients with LSS have been reported to have osteoporosis and a high risk of fractures due to falls [15, 16]. No effective postoperative fall risk assessments or fall prevention measures have been performed for patients with LSS because only a few studies have evaluated falls in these patients. Identifying preoperative predictors of postoperative falls may contribute to the prevention of postoperative falls by identifying patients at high risk of falling and guiding them to appropriate preoperative interventions. Thus, we conducted this study to evaluate the incidence and preoperative predictors of falls within 12 months of surgery in patients with LSS.

## Methods

### Participants

**Fig. 1:**
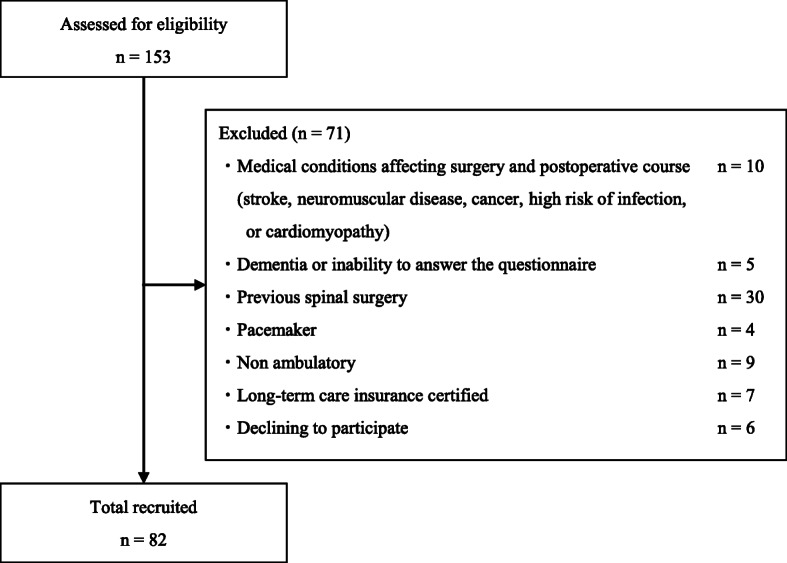
Participant selection flowchart

In this prospective single-center study, consecutive patients with both clinically and radiologically defined LSS underwent surgical treatment at one or two levels (decompression, or posterolateral or transforaminal lumbar interbody fusion) between October 2015 and April 2018. Exclusion criteria consisted of (1) medical conditions such as stroke, neuromuscular disease, cancer, high risk of infection, or cardiovascular disease that researchers have determined to affect surgery or might lead to an unusual postoperative course; (2) dementia or inability to complete the questionnaire independently; (3) previous spine surgery; (4) pacemaker use; (5) non-ambulatory status; and (6) need for long-term care insurance certified by the Ministry of Health, Labour and Welfare. There were 82 (41 male and 41 female) patients included in this study (Fig. 1). All participants provided written informed consent. The study was approved by the local ethics committee of the Tottori University Faculty of Medicine on 31 August 2015 (No. 1508B013).

### Baseline data

As part of the preoperative evaluation that occurred between hospital admission and surgery, demographic data were collected. In addition, participants completed questionnaires and functional tests described below. The results of the preoperative evaluation were used as baseline data.

### Demographic and clinical information

Characteristics such as age, sex, body height, body weight, body mass index (BMI), symptom duration, neurological findings such as deep tendon reflexes (patellar tendon reflex and Achilles tendon reflex) and sensory disturbance, history of fragility fracture, and comorbidities such as hypertension, hyperlipidemia, and diabetes were extracted from medical records. For deep tendon reflexes, the results on the symptomatic side were used. Deep tendon reflexes were categorized as 0 for absent, 1 for diminished, 2 for normal, and 3 for increased. A history of previous falls in the 12 months before the baseline assessment was recorded.

### Data collection

Leg pain and back pain were evaluated using a numerical rating scale (NRS) [17].

Clinical data were evaluated using the Japanese Orthopaedic Association (JOA) score for low back pain [18].

Anxiety and depression were measured using the 14-item Hospital Anxiety and Depression Scale (HADS) [19]. There are seven items each for anxiety and depression. Items are scored from 0 to 3, with higher scores indicating greater anxiety (HADS-anxiety) or depression (HADS-depression). Subscale scores range from 0 to 21. The reliability and validity of the Japanese version of this scale have been verified [20, 21].

### Motor function and structure evaluation

Lower extremity muscle strength was assessed using manual muscle testing (MMT) [22]. The target muscles were the tibialis anterior, extensor hallucis longus, and extensor digitorum longus. If muscle weakness occurred on both sides, the result on the side with greater muscle weakness was considered representative.

Walking speed was assessed using 10-m walking test. The time required to walk 10 m at the participant’s normal speed was used to calculate walking speed. Normal walking speed is an evaluation metric that has been shown to be reliable [23].

Handgrip strength was measured using a T.K.K. 5401 dynamometer (Takei Scientific Instruments Co., Ltd., Niigata, Japan). The highest score was used in the analysis [24].

Muscle mass was measured using bioelectrical impedance analysis (BIA) with an MC-780A body composition analyzer (Tanita Corp., Tokyo, Japan) [25]. Appendicular lean mass was derived as the sum of upper and lower extremity lean mass. Skeletal muscle mass index (SMI) was calculated by dividing appendicular lean mass by the square of body height in meters (kg/m^2^).

Details of these evaluations are described in our previous article [26].

### Sarcopenia and low muscle mass assessment

The Asian Working Group for Sarcopenia 2019 criteria [27] were used to define sarcopenia. We defined sarcopenia as reduced muscle strength (grip strength < 28 kg in men and < 18 kg in women) or reduced physical function (walking speed < 1.0 m/sec) and low skeletal muscle mass (SMI < 7.0 kg/m^2^ in men and < 5.7 kg/m^2^ in women). In this study, we defined low muscle mass as less than 7.0 kg/m^2^ in men and 5.8 kg/m^2^ in women, based on a previous report using the BIA method in Japanese participants [28].

### Fall assessment

A fall was defined as an event in which the participant unintentionally came to rest on a lower level or the floor that was not the result of a major intrinsic event. The follow-up period was 12 months [29]. The incidence of falls was assessed based on participants’ answers to questions about falls during outpatient visits at 3, 6, and 12 months after surgery. Nine months after surgery, we sent a follow-up self-administered questionnaire to assess fall history.

### Statistical analysis

Participants were classified as either non-fallers or fallers, who could have experienced one or more falls, based on data gathered prospectively during the 12 months after surgery.

Data are presented as medians and interquartile ranges. In this study, the following analyses were conducted to evaluate preoperative predictors of postoperative falls. The Mann-Whitney U test was used for continuous variables and ordinal variables, and the chi-square test for nominal variables to compare baseline data between groups. The Wilcoxon signed-rank test was used to compare preoperative and 12-month postoperative outcomes. JOA score, HADS scores, walking speed, handgrip strength, and SMI were treated as continuous variables. NRS and MMT were treated as ordinal variables. Sarcopenia and low muscle mass were treated as nominal variables.

Binomial logistic regression was used to investigate preoperative factors associated with falls during the 12 months after surgery. Variables that were significantly different between groups were considered independent variables because preoperative factors that are predictive of postoperative falls in patients with LSS are unknown. The Hosmer-Lemeshow test was used to evaluate model fit. All data were analyzed using SPSS statistical software (version 24 for Windows; IBM Co., Armonk, NY, USA); *P* values less than 0.05 were considered statistically significant.

## Results

Seventy-four patients (90.2%) completed the 12-month postoperative follow-up. Eight patients were lost to follow-up: five patients did not attend follow-up outpatient visits, two patients developed cancer, and one patient became paralyzed after surgery. Mean procedure time was 140.1 ± 62.3 min, mean length of stay was 18.0 ± 4.7 days, and mean postoperative rehabilitation time was 141.6 ± 59.7 min.

Falls occurred in 24 (32.4%) participants: 10 experienced a fall by 3 months after surgery, 9 by 6 months after surgery, and 5 by 12 months after surgery. Of the participants who fell, 8 (33.3%) had one fall and 16 (66.7%) had two or more falls. Table 1 shows the baseline data of fallers versus non-fallers. On average, participants were admitted to the hospital 3.4 ± 2.3 days before surgery and baseline data were collected 1.7 ± 1.2 days before surgery. Fallers included a significantly higher proportion of females and had a history of falls compared with non-fallers.

**Table 1:** Baseline data

	All (*n* = 74)	Non-fallers (*n* = 50)	Fallers (*n* = 24)	*P*
Age (years)	69.0 (63.0–76.0)	68.0 (63.0–76.0)	73.0 (67.3–76.8)	0.070
Sex (male/female)	36/38	29/21	7/17	0.020
Height (cm)	158.3 (152.0–163.6)	159.3 (153.0–164.7)	156.9 (150.1–163.2)	0.072
Weight (kg)	60.5 (51.6–68.2)	62.7 (53.3–68.7)	54.5 (51.0–63.8)	0.079
BMI (kg/m^2^)	24.0 (22.5–26.2)	24.2 (22.5–26.2)	23.0 (22.4–25.5)	0.255
Symptom duration (months)	11.0 (6.0–54.0)	11.0 (6.0–50.0)	10.0 (4.0–57.0)	0.572
Patellar tendon reflex	2.0 (1.0–2.0)	2.0 (1.0–2.0)	2.0 (1.0–2.0)	0.820
Achilles tendon reflex	1.0 (1.0–2.0)	1.0 (1.0–2.0)	1.0 (0.3–1.0)	0.369
Sensory disturbance (%)	44.6	40.0	54.2	0.251
Fragility fracture (%)	1.4	0.0	4.2	0.146
Comorbidities				
Hypertension (%)	48.6	46.0	54.2	0.511
Dyslipidemia (%)	23.0	20.0	29.2	0.380
Diabetes (%)	14.9	10.0	25.0	0.090
Previous fall (%)	36.5	26.0	58.3	0.007
NRS score for leg pain	5.0 (3.0–7.0)	4.0 (2.8–7.0)	7.0 (4.3–8.8)	0.061
NRS score for back pain	5.0 (3.0–7.0)	4.0 (2.8–7.0)	5.0 (4.3–7.0)	0.290
JOA score	16.0 (12.0–18.0)	16.5 (13.0–20.0)	15.0 (11.0–17.0)	0.038
HADS-anxiety score	5.0 (2.8–8.0)	5.0 (2.0–7.0)	6.0 (3.0–9.0)	0.182
HADS-depression score	5.5 (3.0–8.0)	4.0 (2.0–7.3)	7.0 (4.0–9.0)	0.007
Muscle weakness				
Tibialis anterior	5.0 (5.0–5.0)	5.0 (5.0–5.0)	5.0 (4.0–5.0)	0.017
Extensor hallucis longus	5.0 (4.0–5.0)	5.0 (4.0–5.0)	5.0 (4.0–5.0)	0.138
Extensor digitorum longus	5.0 (5.0–5.0)	5.0 (5.0–5.0)	5.0 (5.0–5.0)	0.228
Walking speed (m/sec)	1.01 (0.78–1.17)	1.05 (0.87–1.20)	0.86 (0.58–1.08)	0.025
Grip strength (kg)	27.6 (22.3–36.1)	31.0 (23.5–38.2)	24.2 (17.9–29.6)	0.005
SMI (kg/m^2^)	6.96 (6.03–7.86)	7.22 (6.14–8.27)	6.40 (5.67–7.43)	0.020
Sarcopenia (%)	10.8	6.0	20.8	0.054
Low muscle mass (%)	20.3	10.0	41.7	0.002

Data are presented as medians (interquartile range) or percentage

*BMI* Body mass index, *NRS* Numerical rating scale, *JOA* Japanese Orthopaedic Association, *HADS* Hospital Anxiety and Depression Scale, *MMT* manual muscle testing, *SMI* Skeletal muscle mass index

Patellar and Achilles tendon reflexes were categorized as 0 for absent, 1 for diminished, 2 for normal, and 3 for increased

Fallers had a significantly lower JOA score and a higher HADS-depression score compared to non-fallers. Fallers had significantly lower muscle strength in the tibialis anterior, gait speed, grip strength, and SMI. They had a higher prevalence of low muscle mass compared with non-fallers.

Table 2 shows fallers and non-fallers stratified by surgical treatment. There were no significant differences by surgery type (*P* = 0.967) and surgical intervertebral level (*P* = 0.638) among fallers and non-fallers. The rate of fusion surgery (posterolateral or transforaminal lumbar interbody fusion) among fallers and non-fallers was 62 and 62.5%, respectively.

**Table 2:** Surgical treatment

	All (*n* = 74)	Non-fallers(*n* = 50)	Fallers(*n* = 24)
Decompression	28	19	9
1 level	(19)	(14)	(5)
2 levels	(9)	(5)	(4)
Fusion	46	31	15
1 level	(27)	(18)	(9)
2 levels	(19)	(13)	(6)

Table 3 shows a comparison of preoperative and 12-month postoperative outcomes. NRS score for leg pain, NRS score for back pain, JOA score, and walking speed improved significantly after surgery.

**Table 3:** Comparison of preoperative and 12-month postoperative outcomes

	Preoperative (*n* = 74)	Postoperative (*n* = 74)	*P*
NRS score for leg pain	5.0 (3.0–7.0)	1.0 (0.0–4.0)	< 0.001
NRS score for back pain	5.0 (3.0–7.0)	1.0 (0.0–3.0)	< 0.001
JOA score	16.0 (12.0–18.0)	22.0 (17.0–26.0)	< 0.001
Walking speed (m/sec)	1.01 (0.78–1.17)	1.23 (1.02–1.36)	< 0.001

Data are presented as medians (interquartile range)

*NRS* Numerical rating scale, *JOA* Japanese Orthopaedic Association

Table 4 shows the binomial logistic regression results. Associations between falls by 12 months after surgery and the following independent variables were investigated: age, sex, previous fall history, JOA score, HADS-depression score, muscle weakness in the tibialis anterior, walking speed, and low muscle mass. Grip strength was not considered an independent variable because it is strongly associated with sex. The presence of low muscle mass was significantly predictive of falls by 12 months after surgery (odds ratio, 4.46; 95% confidence interval, 1.02–19.63). The result of the Hosmer-Lemeshow test was *P* = 0.058.

**Table 4:** Binomial logistic regression analysis of factors associated with falls

Variable	OR	95% CI			*P*
Age	1.03	0.95	–	1.12	0.455
Sex	2.98	0.77	–	11.50	0.113
Previous fall	2.61	0.71	–	9.53	0.147
JOA score	0.88	0.74		1.06	0.183
HADS-depression	1.19	0.98	–	1.44	0.079
Muscle weakness (tibialis anterior)	0.68	0.40	–	1.17	0.164
Walking speed	5.18	0.26	–	104.18	0.283
Low muscle mass	4.46	1.02	–	19.63	0.048

*OR* Odds ratio, *CI* Confidence interval, *JOA* Japanese Orthopaedic Association, *HADS* Hospital Anxiety and Depression Scale

## Discussion

This study prospectively investigated the incidence and predictors of falls within 12 months of surgery in patients with LSS. In this study, the incidence of falls within 12 months of surgery in patients with LSS was 32.4%. Multivariate analysis showed that preoperative low muscle mass is a predictor of postoperative falls. A previous study reported that the annual incidence of falls among older individuals in the general population was 10–20% [30]. Other studies reported the annual incidence as 32.9% in patients who have received total knee replacement [31], 50.0% in patients with osteoporosis [2, 3], and 45.0% in patients with hip osteoarthritis [32]. Therefore, the incidence of falls in patients with LSS after surgery in this study was higher than the incidence among older individuals in the general population and similar to the incidence reported in patients with other musculoskeletal disorders.

A univariate analysis showed that compared with non-fallers, fallers included a significantly higher percentage of women and patients with previous falls as well as a higher preoperative HADS-depression score before surgery. In addition, motor function as assessed by JOA score, tibialis anterior muscle strength, gait speed, and grip strength was lower among fallers. A previous study of older individuals in the general population reported that women have a higher risk of falls than men [33]; the present study of patients with LSS who underwent surgery had consistent findings. The lower grip strength in fallers might be due to the higher percentage of women in the faller group. In addition, findings regarding fall history and depression as well as a decline in motor function based on gait speed and preoperative muscle weakness among fallers in the present study were consistent with those observed in a previous study of community-dwelling older people [9], suggesting that they can be predictors of future falls in patients with LSS.

One strength of this study is that the multivariate analysis, which adjusted for risk factors of falls, showed that a decline in muscle mass is an independent predictor of falls. Although a previous study had suggested an association between a decline in muscle mass and falls in community-dwelling older people [34], there are no similar reports in patients with LSS. Muscle mass decreases with age, as seen in patients with sarcopenia [35]. Furthermore, levels of daily physical activities were likely to be impaired in patients in the faller group because they had significantly lower preoperative JOA scores than patients in the non-faller group. It has been reported that impaired physical activity levels also lead to a decline in muscle mass [35]. Thus, a combination of aging and impaired daily activity levels might have contributed to the significant decline in muscle mass among fallers. A previous study reported that SMI is negatively correlated with pelvic tilt in patients with LSS [36]. Another study reported that poor sagittal plane alignment of the spine, such as pelvic tilt, is a risk for falls [6]. Therefore, the results of the present study suggest that a decline in muscle mass contributed to poor sagittal plane alignment of the spine and consequently increased the risk of falls in patients with LSS. Notably, in this study, the multivariate analysis showed that a decline in muscle mass is a predictor of future falls, instead of factors related to preoperative motor function such as gait speed and lower extremity muscle strength or psychological state such as depression. This result may be related to previous findings that impaired motor function, as shown by slower gait speed and lower extremity muscle weakness, and associated depression could be alleviated by surgery [37–39] while muscle mass could not be increased with surgery [40]. Therefore, assessments to predict falls in patients with LSS should focus on a decline in muscle mass, which can be affected by aging and preoperative physical activity levels, rather than on slower gait speed, lower extremity muscle weakness, and depression which can be alleviated by surgery. Exercise therapy, such as resistance training, that is effective for increasing muscle mass [41] may be useful as preoperative rehabilitation for patients with LSS as postoperative fall prevention.

This study has some limitations. First, the sample size was small; however, the multivariate model had good fit statistics and it was presumed that the small sample size did not significantly affect the results. The power of the post hoc test based on low muscle mass percentage was 83.7%. The second limitation was recall bias. It has been reported that self-administered questionnaire survey responses are affected by the respondent’s age and cognitive function [42]. In this study, therefore, we conducted the questionnaire survey on falls every 3 months during the 1-year study period to reduce the influence of recall bias. The third limitation was a lack of external validity. Generalizing the results of this study to populations in urban regions might not be applicable because this study was conducted in a small rural hospital; however, they may be partially generalizable to such populations because the characteristics of the participant of this study were similar to those of previous studies [6, 7]. The fourth limitation is that the interpretation of the results is limited because this study is not an interventional study. Therefore, in the future, it is necessary to verify the effect of fall prevention interventions on muscle mass in a randomized controlled trial.

## Conclusions

This study investigated preoperative predictors of falls within 12 months of surgery in patients with LSS. The results suggest that the incidence of falls after surgery is high and preoperative low muscle mass may be a predictor of postoperative falls in patients with LSS. Therefore, in patients with LSS, it may be important to assess postoperative fall risk based on various perspectives, including preoperative muscle mass.

## Data Availability

The datasets created during and/or analyzed during the current study are available from the corresponding author on reasonable request.

## Abbreviations

BIA: Bioelectrical impedance analysis
BMI: Body mass index
HADS: Hospital Anxiety and Depression Scale
JOA: Japanese Orthopaedic Association
LSS: Lumbar spinal stenosis
MMT: Manual muscle testing
NRS: Numerical rating scale
SMI: Skeletal muscle mass index
